# *Anopheles gambiae*: historical population decline associated with regional distribution of insecticide-treated bed nets in western Nyanza Province, Kenya

**DOI:** 10.1186/1475-2875-9-62

**Published:** 2010-02-26

**Authors:** M Nabie Bayoh, Derrick K Mathias, Maurice R Odiere, Francis M Mutuku, Luna Kamau, John E Gimnig, John M Vulule, William A Hawley, Mary J Hamel, Edward D Walker

**Affiliations:** 1 Centre for Global Health Research, Kenya Medical Research Institute, PO Box 1578, Kisumu, Kenya; 2 Centers for Disease Control and Prevention, PO Box 1578, Kisumu, Kenya; 3 Centre for Biotechnology Research and Development, Kenya Medical Research Institute, Nairobi, Kenya; 4 Division of Parasitic Diseases, Centers for Disease Control and Prevention, Atlanta, GA, 4770 Buford Hwy., Mailstop F-42, Atlanta GA 30341, USA; 5 Department of Microbiology and Molecular Genetics, Michigan State University, East Lansing, MI, 48824, USA

## Abstract

**Background:**

High coverage of insecticide-treated bed nets in Asembo and low coverage in Seme, two adjacent communities in western Nyanza Province, Kenya; followed by expanded coverage of bed nets in Seme, as the Kenya national malaria programme rolled out; provided a natural experiment for quantification of changes in relative abundance of two primary malaria vectors in this holoendemic region. Both belong to the *Anopheles gambiae sensu lato (s.l.) *species complex, namely *A. gambiae sensu stricto (s.s.) *and *Anopheles arabiensis*. Historically, the former species was proportionately dominant in indoor resting collections of females.

**Methods:**

Data of the relative abundance of adult *A. gambiae s.s. *and *A. arabiensis *sampled from inside houses were obtained from the literature from 1970 to 2002 for sites west of Kisumu, Kenya, to the region of Asembo ca. 50 km from the city. A sampling transect was established from Asembo (where bed net use was high due to presence of a managed bed net distribution programme) eastward to Seme, where no bed net programme was in place. Adults of *A. gambiae s.l. *were sampled from inside houses along the transect from 2003 to 2009, as were larvae from nearby aquatic habitats, providing data over a nearly 40 year period of the relative abundance of the two species. Relative proportions of *A. gambiae s.s. *and *A. arabiensis *were determined for each stage by identifying species by the polymerase chain reaction method. Household bed net ownership was measured with surveys during mosquito collections. Data of blood host choice, parity rate, and infection rate for *Plasmodium falciparum *in *A. gambiae s.s. *and *A. arabiensis *were obtained for a sample from Asembo and Seme from 2005.

**Results:**

*Anopheles gambiae s.s. *adult females from indoor collections predominated from 1970 to 1998 (ca. 85%). Beginning in 1999, *A. gambiae *s.s decreased proportionately relative to *A. arabiensis*, then precipitously declined to rarity coincident with increased bed net ownership as national bed net distribution programmes commenced in 2004 and 2006. By 2009, *A. gambiae s.s. *comprised proportionately ca. 1% of indoor collections and *A. arabiensis *99%. In Seme compared to Asembo in 2003, proportionately more larvae were *A. gambiae s.s.*, larval density was higher, and more larval habitats were occupied. As bed net use rose in Seme, the proportion of *A. gambiae *larvae declined as well. These trends continued to 2009. Parity and malaria infection rates were lower in both species in Asembo (high bed net use) compared to Seme (low bed net use), but host choice did not vary within species in both communities (predominantly cattle for *A. arabiensis*, humans for *A. gambiae s.s.*).

**Conclusions:**

A marked decline of the *A. gambiae s.s. *population occurred as household ownership of bed nets rose in a region of western Kenya over a 10 year period. The increased bed net coverage likely caused a mass effect on the composition of the *A. gambiae s.l. *species complex, resulting in the observed proportionate increase in *A. arabiensis *compared to its closely related sibling species, *A. gambiae s.s. *These observations are important in evaluating the process of regional malaria elimination, which requires sustained vector control as a primary intervention.

## Background

Recent progress in reducing malaria morbidity and mortality in Africa is founded upon expanded coverage of insecticide-treated bed nets (hereafter, bed nets), indoor residual spraying, and combination drug therapy [1]. For this progress to translate into the ambitious goal of malaria elimination, most agree that vector control has a central role [1-3]. Yet, there is an incomplete understanding of how these insecticide-based interventions affect vector populations during long-term implementation, even though a long-term perspective (10+ years) is required to comprehend well the relationship between effectiveness of anti-vector measures and prevalence of malaria infection in humans [4].

Vector populations can respond behaviourally, numerically, or evolutionarily to insecticides implemented against them in malaria control programmes. With regard to behaviour, females of some *Anopheles *species show elevated activity due to the excitation effects of the active ingredients in some insecticide formulations of indoor residual sprays or insecticide-treated bed nets, resulting in their movement away from the sprayed walls or treated nets, with or without having obtained a human blood meal [5-8]. With regard to numeric responses to these interventions, malaria vector populations typically diminish in density and have reduced longevity [9-11]. For example, *Anopheles gambiae s.l. *and *Anopheles funestus *population density declined markedly in a randomized evaluation trial of permethrin-treated bed nets in treatment compared to control villages in western Kenya [12], an effect which persisted for three years after the trial ended and after all villagers were given treated nets that were retreated at 6-9 month intervals [13]. Evolutionary responses typically involve changes in phenotypic sensitivity to the insecticides being used, when alleles associated with reduced target site sensitivity or enhanced metabolic detoxification increase in frequency [14].

In the present study, research was focused on the population numeric responses of *Anopheles gambiae s.l. *mosquitoes to long-term implementation of insecticide-treated bed nets in western Nyanza Province, Kenya. This species complex contains six species whose members are indistinguishable morphologically but which differ in certain behavioural and ecological attributes that are important to their vectorial capacity for malaria and for sampling [15-18]. *Anopheles gambiae s.s. *and *Anopheles arabiensis *are the two most common members of this complex and the only two found in western Kenya; *A. gambiae s.s. *feeds mostly on humans, whereas *A. arabiensis *feeds mostly on cattle and other animals, less so on humans, making it a less efficient but still capable malaria vector [17,18].

The region where the research reported here was conducted, in the Asembo Bay area of Nyanza province in western Kenya, has been an area of active research on effectiveness of insecticide treated bed nets in reducing malaria transmission, and malaria-related morbidity and mortality in people [13,19-21]. In a randomized trial of the effectiveness of permethrin-treated bed nets on malaria infection and transmission commencing in late 1996, all houses in selected villages in Asembo received bed nets, whereas another set received no nets and served as controls; in 1999, houses in all villages received them, leading to high coverage of bed nets there that has been maintained to 2007 through the provision of free retreatment services and periodic net replacement [13,20]. The original trial showed that indoor density of *Anopheles *vectors of malaria diminished substantially, villagers' health improved, and child mortality declined [19,21]. These trends were sustained for four years after the trial ended and as net coverage was sustained [13,20]. Populations of *A. funestus *diminished to negligible levels, when bed nets were used at high coverage in the trial in western Kenya, whilst mosquitoes of the *A. gambiae s.l. *complex persisted as transmission declined [19]. There was no bed net distribution programme in a nearby and identical community called Seme, bordering Asembo to the east [13]. In samples of adult female mosquitoes taken from inside houses between 1999 and 2002, Lindblade *et al *[13] observed that the proportion of *A. gambiae s.s. *was significantly less in Asembo (51.2%) compared to Seme (77.4%), suggesting that the greater number of permethrin-treated bed nets in Asembo was disproportionately affecting populations of the former species. Building upon this observation, we postulated that populations of *A. gambiae s.s. *would decline when bed nets were owned and used at high rates, compared to the local sibling species, *A. arabiensis*. The increasing and well-documented patterns of bed net coverage in Asembo and Seme allowed a test of this hypothesis by measuring changes in numbers of adult and larval mosquitoes of both species over several years. Further, historical data were obtained to examine multi-decadal trends in changes in the proportions of these two species as the national malaria campaign in Kenya resulted in increases in bed net ownership regionally.

## Methods

### Study area

**Figure 1:**
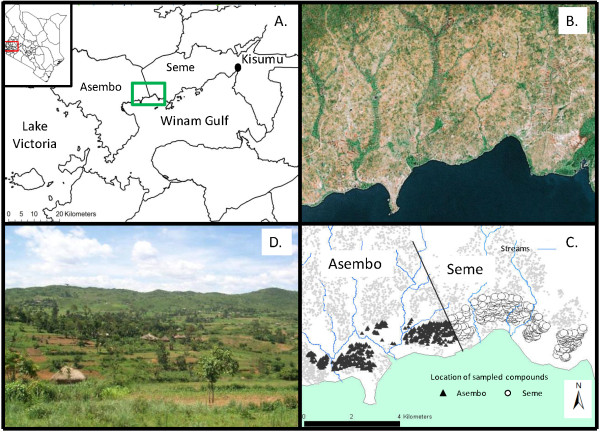
**A. Location of study site in western Kenya**. Small insert map shows Kenya; red box indicates study region in western Nyanza province. Locations of Asembo and Seme are indicated. Green box indicates sampling transect from 2003 to 2009 (as in B and C). B. Google Earth satellite image of transect region in the Asembo and Seme study area, showing lake plain topography, numerous stream courses, and rural agricultural landscape. C. 12 km sampling transect from Asembo into Seme, showing location of mapped, sampled compounds in 2003. D. Photograph of study area facing north, showing housing compounds, surrounding farmland, and topography.

This study was conducted in Kisumu and Bondo districts in western Nyanza Province, Kenya, extending west of the city of Kisumu to the community of Asembo (Figure 1A). Studies on effects of indoor residual spraying with fenitrothion on malaria vectors were conducted in this area in the early 1970s [22,23]. A randomized, controlled trial of the effect of permethrin-treated, conventional bed nets on vector populations and malaria transmission was conducted in the same area in the late 1990s [21]. That trial was extended to a managed malaria control programme involving retreatment of nets in organized community campaigns at 6-9 month intervals with permethrin until 2002, then with alphacypermethrin until 2007 (20; M.N. Bayoh, M. Hamel, and J. Gimnig, unpublished). The present research was conducted in part of that original bed net study site (Asembo, Rarieda Division, Bondo District, Nyanza Province) and in an otherwise similar area not included in that trial but bordering the eastern part of it called Seme (Kombewa Division, Kisumu District, Nyanza Province) (Figure 1A) [13,20]. Permethrin-treated, conventional bed nets were distributed to half (late 1996) and then all (early 1999) of the residents of Asembo, providing nearly 100% household ownership and high rates of nightly use [20,24,25]. By contrast, there was no bed net distribution programme in Seme, thus coverage was very low (< 5% of houses; [13]) until the initiation of distribution of long-lasting, insecticide treated bed nets by the Kenyan Ministry of Health to pregnant women and children < 5 years beginning in 2004 (at subsidized price) followed by a mass campaign targeting children < 5 years conducted in 2006 (free to mothers of children under five years of age) http://www.nmcp.or.ke.

### Contemporary sampling

**Figure 2:**
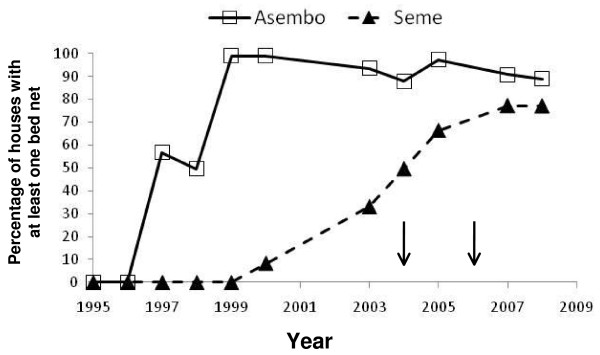
**Household ownership of bed nets, defined as at least one per house, of any bed net type and whether treated or untreated, in Asembo (site of bed net trial in the late 1990s) and Seme (no bed net trial) from 1995 through 2008**. Arrows indicate the initiation of subsidized national distribution of bed nets through health clinics to pregnant women and children < 5 years (2004) and a mass campaign during which 3.4 million nets were distributed for free to children < 5 years in all endemic regions of Kenya (2006).

In 2003, larval mosquitoes were sampled quantitatively using area samplers [26,27] from habitats situated within 200 m of all housing compounds along a 1 × 12 km transect established north of Lake Victoria within Asembo and Seme (Figure 2). Habitats were sampled twice (April-May, and June) in 2003. Adult mosquitoes were sampled inside houses with pyrethrum spray collections [19] in June. From 2006-2009, larval mosquitoes were sampled qualitatively (i.e., without regard to unit area of habitat) using dippers and pipettes along a similar transect during April-June of each year, solely for the purpose of determining the proportions of larvae that were *A. gambiae s.s. *and *A. arabiensis*. Adult females were sampled from 20 houses in four discrete sites each along the sampling transect in 2005 [28]. The sampling transect was progressively increased in area from 12, 20, 48, 80, 80, and 80 km^2 ^for the years 2003, 2005, 2006, 2007, 2008, and 2009, respectively, with equal areas sampled in Asembo and Seme, and minimally 100 houses sampled. The reason was that *A. gambiae s.s*. had become progressively rarer and mosquitoes overall less abundant, requiring greater sampling area to find individuals of this species. Adult females were sampled in 2005, 2007, 2008, and 2009 from inside houses using either aspiration by hand or the pyrethrum spray catch method for purposes of determining the proportions of the two species as well. *Anopheles gambiae s.l. *were identified by PCR [29,30], as *A. gambiae s.s. *or *A. arabiensis*. Adult mosquitoes sampled from subsets of compounds along the transect in Asembo and Seme in 2005 [28] were dissected to determine parity [31] analysed for host blood using an ELISA procedure with anti-human and anti-bovine reagents [32], and tested for salivary gland infection for *Plasmodium falciparum *sporozoites [33].

### Bed net ownership

Bed net ownership in Asembo and Seme was assessed during indoor adult mosquito collections by noting presence or absence of a net, or from cross-sectional surveys conducted by the Kenya Medical Research Institute and US Centers for Disease Control and Prevention. Additional data of bed net ownership from 2003 for Seme were provided by Dr. Mark Polhemus of the Walter Reed Army Institute of Research, Kisumu, Kenya.

### Historical data

Data of the abundance and ratio of adult, female *A. gambiae s.s. *and *A. arabiensis *were obtained from indoor collections from nine published studies conducted from 1970 to 2002 in villages in this area (see Additional File 1). Prior to the development of molecular based methods [29,30], the two species were distinguished as "species A" and "species B" by preparation of polytene chromosomes and microscopic examination of banding patterns on chromosome X [18]. Data from these studies were only utilized from locations situated west of the city of Kisumu to Asembo (Figure 2A), so that data of adult females obtained from 2003 to 2009 (see below) would be comparable. Data from other collection sites outside of this lake plain sampling zone were purposefully excluded to remain consistent with the local conditions and mosquito populations under study.

### Data analysis

The effect of sampling location on abundance was assessed by Poisson regression. The proportion of *A. gambiae s.s. *to *A. arabiensis *by distance from the border separating Asembo and Seme was analyzed using logistic regression in the GENMOD procedure, adjusting for clustering and repeated measures. For all transect analyses, tests for trends were done by creating continuous categorical variables based upon distance from the border between Asembo and Seme. Collection sites were grouped into 2 km categories with the sites furthest inside Asembo assigned a 1 and those furthest inside Seme assigned a 6. The outputs of the logistic regression analyses, therefore, show the change in the odds that a mosquito would be identified as *A. gambiae s.s. *for every 2 km, as one moves east from the center of Asembo. For the 2005-2009 samples, data of number of adults and larvae from 2003 provided expected values to test for the effects of increased ITN coverage using χ^2 ^goodness of fit tests. If coverage had no effect on species composition, then the null hypothesis was that frequencies in Seme should not differ between 2003 and those years.

## Results

### Changes in bed net ownership over time

In Asembo, the percentage of houses with at least one bed net was >95% in 1999 and remained high, although slightly declined, through 2008. Coverage was initially low in Seme, but rose to levels approaching those observed in Asembo by 2008 (Figure 2).

### Population dynamics of *A. gambiae s.s. *and *A. arabiensis*

**Figure 3:**
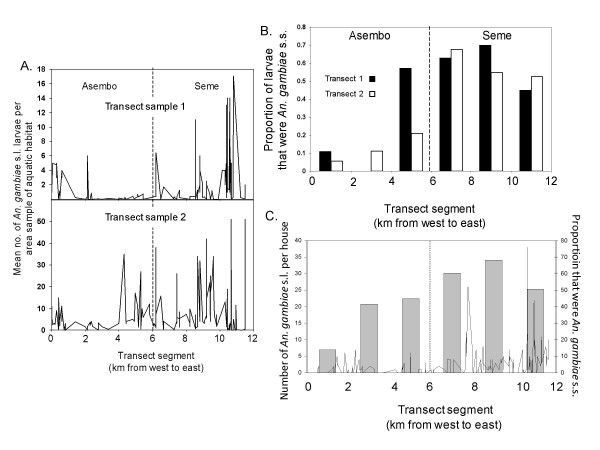
***Anopheles gambiae *s.l. larval and adult population density in 2003**. A. Density of *A. gambiae *s.l. larvae (N = 1,719) in habitats sampled twice along a 12 km transect from Asembo to Seme. B. Proportion of *A. gambiae *s.l. larvae that were *A. gambiae *s.s. (as opposed to *A. arabiensis*). C. Density of adult, female *A. gambiae *s.l. inside houses (line), and proportion of them identified by PCR as *A. gambiae *s.s. (bars), along the Asembo-Seme transect.

In 2003, when bed net coverage was high in Asembo but low in Seme (Figure 2), Seme had a higher proportion of larval habitats with *A. gambiae *s.l. larvae (73.8%, N = 187 habitats and 1,361 larvae) than Asembo (58.8%, N = 102 habitats and 358 larvae). Further, density of *A. gambiae *s.l. larvae was lower in Asembo than in Seme in each of the two transect sweeps. It was higher overall in the second transect sample compared to the first, likely representing population growth during the course of the rainy season that year (Figure 3A). Density increased significantly with transect sampling position from Asembo to Seme in Poisson regression, and was observed for both sampling events (1^st ^transect sample: risk ratio = 1.28, 95% confidence interval = 1.04-1.58, P = 0.006; 2^nd ^transect sample: risk ratio = 1.12, 95% confidence interval = 1.02-1.24, P = 0.028). In the first transect sample, the proportion of *A. gambiae *s.s. larvae relative to *A. arabiensis *was 16.7% in Asembo but 59% in Seme (Figure 3B). Similarly, in the second transect sample, the proportion of *A. gambiae s.s. *relative to *A. arabiensis *was 9.0% in Asembo and 56.6% in Seme. In both transects, within Asembo, the proportion of *A. gambiae *s.s. increased with decreasing distance to the border with Seme (Figure 3B). The probability that an individual *A. gambiae s.l. *was identified as *A. gambiae s.s. *increased significantly (logistic regression) with transect sampling position from Asembo to Seme (1^st ^transect: risk ratio = 1.36, 95% confidence interval = 1.09-1.68, P = 0.006; 2^nd ^transect: risk ratio = 1.77, 95% confidence interval = 1.49-2.10, P < 0.001.). Density of adult female *A. gambiae s.l. *inside houses was lower in Asembo than Seme, and the proportion of them that were *A. gambiae s.s. *was lower within Asembo (27.0%) compared to Seme (58.4%) (Figure 3C). The probability that an individual, female *A. gambiae *s.l. sampled indoors was *A. gambiae s.s. *increased significantly from Asembo to Seme along transect sampling points (logistic regression; risk ratio = 1.27, CI = 1.08-1.49, P = 0.009).

**Figure 4:**
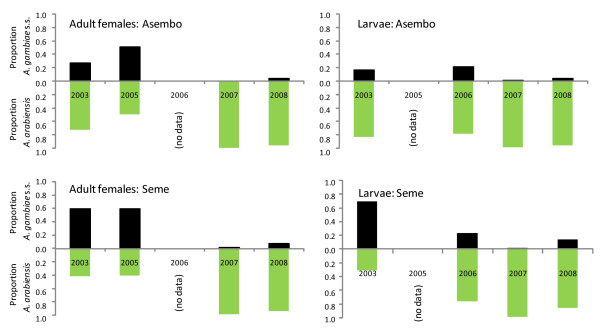
**Proportion of *A. gambiae *s.l. adult females and larvae that were identified as *A. gambiae *s.s. (top, black bars) or *A. arabiensis *(bottom, green bars) from Asembo and Seme in 2003, 2005 (adults only), 2006 (larvae only), 2007, and 2008**.

The relative proportions of *A. gambiae s.s. *and *A. arabiensis *in larval and adult female stages in Asembo and Seme are shown in Figure 4. The proportion of adult females or larvae that was *A. gambiae s.s. *was lower in Asembo compared to Seme initially and then equalized in later years when *A. arabiensis *dominated both stages. Within Seme, the proportion that was *A. gambiae s.s. *decreased markedly from 2003 to 2008 (Figure 4). Species composition of females sampled indoors in Seme did not differ significantly between 2003 and 2005 (χ^2 ^= 0.04, df = 1, P = 0.84).* Anopheles gambiae s.s. *larvae were significantly less abundant in 2006 compared to 2003 (χ^2 ^= 41.8, df = 1, P < 0.0001).* Anopheles gambiae s.s. *larvae and adults were rare in both Asembo and Seme in 2007, when bed net coverage was high in both areas (Figure 4). Of 264 adult specimens identified as *A. gambiae s.l. *from indoor collections in 2007, only two were *A. gambiae s.s. *(both from Seme). The remainder was *A. arabiensis*. Of 3,185 larvae identified to species in the same year, 26 (0.8%) were *A. gambiae s.s.*, equally from Asembo and Seme. Species composition differed significantly between 2003 and 2007 in Seme, with the proportion of *A. gambiae s.s. *being much lower than expected for adults (χ^2 ^= 157.9, df = 1, P < 0.0001) and larvae (χ^2 ^= 746.5, df = 1, P < 0.0001). In 2008, the proportions of larval and adult *A. gambiae s.l. *that were *A. gambiae s.s. *remained low in Asembo, but were higher in Seme compared to 2007 (Figure 4). Of 350 adult specimens identified as *A. gambiae s.l. *from indoor collections, 313 produced PCR amplicons and only 37 (11.8%) were *A. gambiae s.s. *(18 from Seme, 19 from Asembo), the remainder being *A. arabiensis*. Of 497 larvae identified as *A. gambiae s.l.*, 456 produced PCR amplicons; only 41 (9.0%) of these were *A. gambiae s.s. *(eight from Asembo, 33 from Seme). The proportion that was *A. gambiae s.s. *in 2008 was lower than expected compared to 2003 for adults (χ^2 ^= 5.8, df 1, P = 0.02) and larvae (χ^2 ^= 81.1, df 1, P < 0.0001). In 2009, only one of 94 (1.1%) adult, female specimens that reacted in PCR was *A. gambiae s.s.*; the others were *A. arabiensis*. The proportion of *A. gambiae s.s. *was significantly lower in 2009 than in 2003 (χ^2 ^= 54.6, df 1, P < 0.0001). An additional 128 females that were identified morphologically as *A. gambiae s.l. *did not react in PCR; due to a storage problem, no *A. gambiae s.l. *larvae reacted in PCR from 2009 samples, thus, the ratio of *A. gambiae s.s. *and *A. arabiensis *for larvae in that year could not be calculated.

### Parity, host choice, and malaria infection

**Figure 5:**
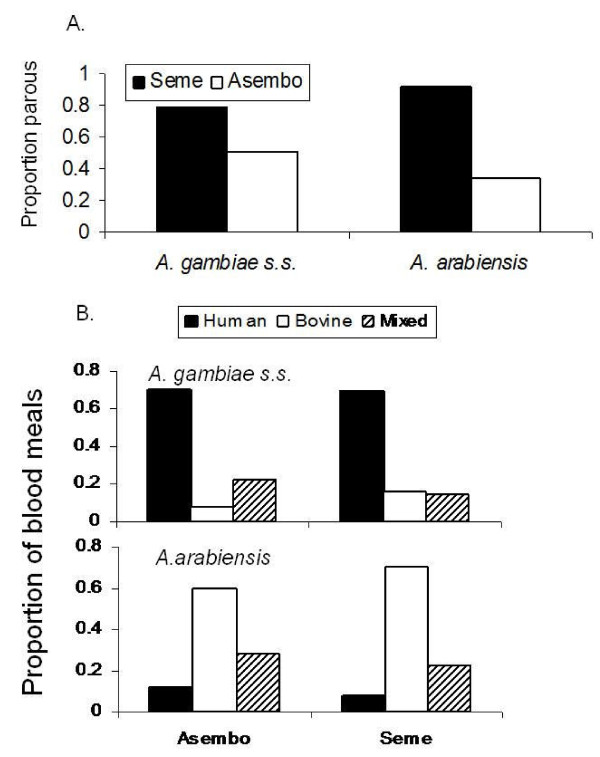
**A. Proportion parous of *A. gambiae *s.s. and *A. arabiensis *in Seme compared to Asembo**. B. Proportion of mosquitoes that had blood fed on humans, cattle, or mixture of both in Asembo and Seme. Nonreactors in blood meal ELISA not shown (42%).

In 2005, the proportion of females in the parous condition was lower in Asembo compared to Seme for both *A. gambiae s.s. *in Asembo (n = 329) and *A. arabiensis *(n = 207) (Figure 5A). Based on ELISA for circumsporozoite protein, *A. gambiae s.l. *had a lower infection rate in Asembo (0.8%, n = 569) than Seme (1.95%, n = 1,331) although these differences were not significant (2 × 2 contingency table, χ^2 ^= 3.23, df = 1, P = 0.07). A total of 149 *A. gambiae s.s. *and 153 *An arabiensis *were tested for blood host (Figure 5B). *Anopheles arabiensis *fed most frequently on bovines (65% of blood meals; 22% mixed bovine/human; 13% human) and *A. gambiae s.s *on humans (70% of blood meals; 21% mixed human/bovine; 9% bovine); there were no significant differences in host choice within species between the two sites (*A. gambiae s.s*.: χ^2 ^= 3.61, df = 2, P = 0.164; *A. arabiensis*: χ^2 ^= 1.66, df = 2, P = 0.436); however, host selection was significantly different between mosquito species without regard to study sites (2 × 3 contingency table, χ^2 ^= 123.4, df = 2, P < 0.0001).

### Rainfall and temperature

**Figure 6:**
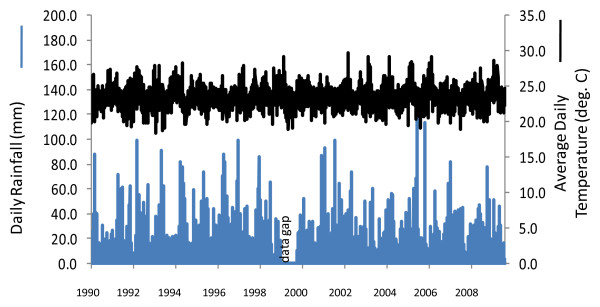
**Mean daily temperature and daily rainfall at Kisumu airport weather station, 1990 to 2009**.

Data of daily rainfall and average daily temperature from 1990 to 2009 in Kisumu airport showed no aberrant trends (Figure 6).

### Historical decline of *A. gambiae s.s*

**Figure 7:**
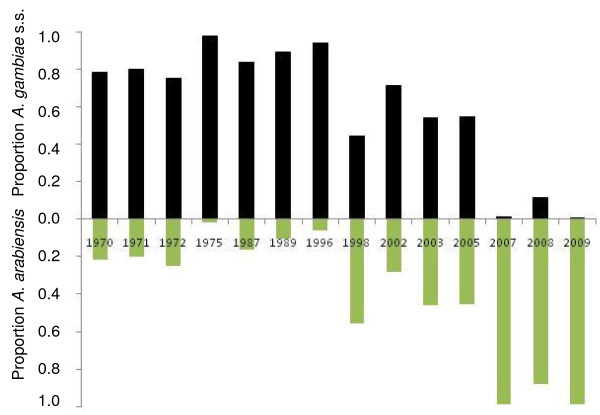
**Proportion of adult female *A. gambiae *s.l. mosquitoes collected west of Kisumu and identified as *A. gambiae *s.s. (top, black bars) or *A. arabiensis *(bottom, green bars)**. Data from 1970 through 2002 are compiled from published data (see Additional File 1), and data from 2003 onwards are from the current study.

The proportion of *A. gambiae s.s. *in samples of females collected indoors, was high relative to *A. arabiensis *from 1970 to 1998, but thereafter declined (Figure 7). By the years 2007-2009, *A. gambiae s.s. *had become relatively uncommon compared to *A. arabiensis*, such that the ratios of the two species virtually reversed during the course of these few decades. In 2009, *A. gambiae s.s. *was only 1.1%.

## Discussion

Historical review of data on the relative proportions of *A. gambiae s.s. *and *A. arabiensis *females sampled indoors from 1970 to 2002, as well as more contemporary data from sampling efforts of larvae and adult females of these two species reported here, showed a decline in the predominance of the former species with a comparative proportionate increase in the latter species (Figures 3, 4, and 7). Any sampling bias would likely be against *A. arabiensis *females in indoor collections due to their relatively reduced likelihood of entering and resting in houses, compared to *A. gambiae s.s. *females [17,18,30] thus differential sampling in favor of *A. arabiensis *is highly unlikely to be an explanation for the trend. As larvae of these species show no habitat segregation in this study area [34,35], changes in larval numbers should accurately reflect population densities and true proportions of the two species, particularly because sampling was done during wet periods, when both species were abundant. The quantitative sampling data from 2003 (Figure 3) and qualitative sampling data to 2008-09 (Figure 4, Figure 7) clearly illustrate a process of gradual extirpation of *A. gambiae s.s. *in the study area, but persistence of *A. arabiensis*. Larval sampling facilitated delineation of this process and should prove useful to others who wish to compare relative changes of the two species under similar conditions.

In Asembo, Seme and regionally, the decline in *A. gambiae s.s. *coincided geographically and temporally with scale-up of national programmes leading to high rates of household ownership (and presumably, use) of bed nets, suggesting that presence of the bed nets in most houses caused the observed population decline. Alternative explanations seem less likely. First, biased sampling, if having an effect, would have worked against the trend. Second, there was no evidence of an environmental or climate change that could have affected the species distributions locally; indeed, temperature and rainfall were consistently within a normal range across a two decade period (Figure 6). More importantly, a recent prediction from ecological niche models based on climate change scenarios was that *A. gambiae s.s. *should increase while *A. arabiensis *should remain stable or decline regionally [36], opposite of what was observed. Third, the changes might be related to differential abundance of cattle and human hosts. However, in a survey conducted before the current study commenced, both hosts were common in both areas, with about 2.5 cattle and 3.6 people per compound in Asembo and 3.8 cattle and 3.2 people per compound in Seme (J. Gimnig, unpublished data). Cattle are commonly husbanded throughout western Kenya, however, this observation suggests that a greater abundance of *A. arabiensis *in Asembo could not be explained by more cattle there compared to Seme. Fourth, a decline in *A. gambiae s.s. *populations in Seme subsequent to programmatic scale-up was predicted, based upon the observations from Lindblade *et al *[13] on proportionate differences of indoor resting, female *A. gambiae s.s. *relative to *A. arabiensis *in Asembo and Seme in 2002, but before the national programme commenced. Results from the present study confirmed this prediction for the prolonged period from 2003-2009, when *A. arabiensis *adults and larvae profoundly outnumbered *A. gambiae s.s*. in Asembo and became proportionately dominant in Seme, in sharp contrast to the historic trend prior to arrival of bed nets in that community and in nearby ones. Finally, the correlation through time between increase in bed net ownership (Figure 2) and decrease in *A. gambiae s.s. *(Figure 4, 7) could be a mere coincidence, an interpretation which seems highly unlikely given the historical dominance of this species in the region west of Kisumu; and given the results of the intensive transect sampling in 2003 over a relatively short distance (12 km) (Figure 3).

The most plausible biological mechanism for our primary result is straightforward: bed nets acted as lethal, human-baited traps or as strong repellency devices for the highly anthropophilic, female *A. gambiae s.s.*, causing their population to crash. Aside from direct mortality, blood feeding inhibition, partially due to the excite-repellency effect of bed nets, could induce mortality through deprivation of blood. Fewer and shorter-lived adult *A. gambiae s.s. *laid fewer eggs in larval habitats, resulting in fewer larvae, reducing larval habitat occupancy and larval density. The decline in *A. gambiae s.s. *populations is provocative on several levels. First, any malaria control programme is imperfect, with some families not receiving bed nets or, if possessing them, not using them nightly or not retreating them regularly. In Asembo, recent observations indicate that of those families owning bed nets, only 77% use them regularly when sleeping (M. Hamel, unpublished data). Thus, ownership does not equate to use. Nets in Asembo were retreated at regular intervals by house to house campaigns through 2003. Thereafter, retreatment was available at central locations at regular intervals and the service was free through 2007, but retreatment rates (not quantified) were certainly never 100% (M.N. Bayoh, M. Hamel, unpublished observations). In Seme, household ownership of nets increased through efforts by the Kenya Ministry of Health yet remained incomplete after the second roll-out (Figure 2). Nonetheless, a massive population decline in a major, anthropophilic vector occurred despite these imperfections. Second, while *A. gambiae s.s. *historically showed considerable flexibility in resting behaviour when confronted with widespread indoor residual spraying [8,10,11,14], results from the present study suggest less flexibility in host choice (Figure 5B). There was no strong blood host shift to non-humans, nor a shift to predominantly outdoor resting [28], in the face of strong pressure from bed nets. Bogh *et al *[37] found that *A. gambiae s.l. *females shifted slightly in host selection away from humans towards cattle when permethrin-treated bed nets were distributed in villages on the Kenya coast. However, the mosquitoes were not identified to sibling species in the complex by PCR, thus any species-specific changes in host selection were not revealed in that study. Third, results reported here are consistent with negligible density-dependent effects influencing *A. gambiae s.s. *population dynamics. This is in contrast to strong density-dependent controls operating in *Aedes *mosquito populations [38], but in agreement with results of field studies of *A. gambiae s.s.*, which demonstrate only moderate density-dependence [39]. Cumulative adult female mortality due to exposure to pyrethroid toxins in bed nets appears not to be buffered by density-dependent modulation in immature stages, where density-independent processes such as disturbance dominate [26], thus the killing effect of bed nets remains strong even as vector densities are driven low. The increase in bed net coverage described here (Figure 2) likely resulted in reductions in survival, total lifetime fecundity, and basic reproductive number of *A. gambiae s.s. *females in the study area cumulatively over many generations. The relatively lower parity rate observed in *A. arabiensis *compared to *A. gambiae s.s. *in Asembo compared to Seme in 2005 might be interpreted as a greater effect of bed nets on the former species, potentially confounding the interpretation of the mechanism of decline of the latter species. However, sampling bias against *A. arabiensis *resting and feeding outside of the peridomestic setting would result in over-sampling of those female *A. arabiensis *affected by bed nets indoors, therefore explaining the apparent discrepancy [17,18].

The decline of an anthropophilic, anopheline mosquito species; and corresponding proportionate rise of a zoophilic one; during malaria vector control has rarely been reported in Africa. In the Pare-Taveta region of northern Tanzania and southeastern Kenya, indoor residual spraying with dieldrin resulted in the near elimination of *A. funestus*, whilst absolute numbers of the closely related but zoophilic species, *Anopheles rivulorum*, increased dramatically [40]. Even though spraying ceased in 1959, *A. funestus *populations and malaria transmission remained suppressed into 1966 [41], demonstrating long-term and vigorous effects of the original programme. The increase in not just proportion, but density of *A. rivulorum*, was unexpected and difficult to explain. *Anopheles funestus *has not been replaced by *A. rivulorum *in the Asembo area, *A. funestus *populations remain very low [27,30], nor did *A. arabiensis *increase in absolute numbers as *A. gambiae s.s. *declined (see Figure 3). In western Kenya, near the Asembo study site, an indoor residual spray programme using fenitrothion resulted in a moderate increase in the proportion of adult *A. arabiensis *compared to *A. gambiae s.s*. [42], but species structure of larval populations did not shift in tandem, and both the intervention and evaluation periods were short-term, not allowing for analysis of long-term effects as done here. In the Garki project in northern Nigeria, there was no observed shift in proportions of the two species after a period of indoor residual spraying, although entomological surveillance was a minor component of that evaluation [43]. In South Africa, where indoor residual spraying was implemented effectively to reduce malaria burden, *A. gambiae s.s*. apparently disappeared whereas the zoophilic species *Anopheles quadriannulatus *(also a member of the *A. gambiae s.l. *complex, but not a malaria vector) persisted, and residual malaria transmission was attributed to *A. arabiensis *[4]. However, these changes were qualitatively documented and no larval data were available for unbiased comparisons of changes in relative species abundance. During the malaria eradication campaign in British Guiana from 1945 to 1949, involving application of DDT on the inner walls of houses as a residual insecticide, larvae and adults of the primary vector (*Anopheles darlingi*) were originally numerous but disappeared, whereas larvae and adults of a zoophilic species, *Anopheles aquasalis*, persisted [44].

The marked decline in *A. gambiae s.s. *in western Kenya has been associated with a simultaneous decline in malaria prevalence from 70% between 1997-1999 [45] to ca. 25% in 2008 in children < 5 years old (M. Hamel, unpublished data). In eastern Kenya, malaria cases declined in children over the time period of 1997 to 2007, with a steep drop after 2004 [46], when the national bed net distribution programme began in earnest; however, O'Meara *et al *[46] could not conclude definitively that the decline in malaria cases was due solely to increased bed net use. By contrast, a similar marked decline in malaria cases in The Gambia over the same time period appeared to be related mainly to increased use of bed nets [47]. However, neither the eastern Kenya nor The Gambia study provided mosquito community composition data to correlate with the declines in malaria in humans. The implication of the research reported here is that sustained, high coverage of bed nets should dramatically reduce malaria transmission by *A. gambiae s.s.*, leaving residual transmission by *A. arabiensis *(see Additional File 2). Indeed, the ratio of *A. gambiae s.s. *to *A. arabiensis *under conditions where both species occur and are transmitting malaria may be a useful relative index of programme effectiveness in places where the former species has been historically the dominant vector, as was the case in parts of southern Africa [4]. With wide coverage of expanded interventions like the one described here, malaria transmission should suffer a precipitous decline mediated through profound effects on vector populations, driving transmission downward and significantly closer to the goal of elimination. Killeen *et al *[48] proposed the need for higher coverage of bed nets when *A. arabiensis *becomes the dominant vector, if elimination is to be achieved; the opportunity to test this hypothesis is now available. The need for alternative control methods for *A. arabiensis *is also apparent.

Recent perspectives on the process of elimination propose a shift from population-based coverage of interventions to a clinical surveillance-based system with expanded drug treatment [3]. Results provided here, by contrast, illustrate the crucial importance of long-term maintenance of high coverage interventions against transmission (such as insecticide-treated bed nets) to ensure continual suppression of key vector species, coupled with long-term vector surveillance as a means of continually assessing programme effectiveness, such as by quantifying species ratios, host selection patterns, and parity rates.

## Competing interests

The authors declare that they have no competing interests.

## Authors' contributions

MNB, JEG, WAH, JMV, and EDW designed the study and wrote the manuscript. MNB, DKM, FM, MRO, JEG, and EDW sampled and processed mosquitoes; DKM, LK, MRO, and EDW identified mosquitoes of the *A. gambiae *s.l. complex with PCR. MJH, JEG, DM, WAH, and JMV obtained data of bed net ownership. All authors read and approved the final manuscript.

## Supplementary Material

Additional file 1 **Historical records of *Anopheles gambiae *s.l. complex from indoor collections in western Kenya**. List of references and summary of historical data used for composition of Figure 7, showing the year of sampling, village names or sample locations, number of houses sampled per location, and number of adult, female mosquitoes in the *Anopheles gambiae *sensu lato complex identified as either *Anopheles gambiae sensu stricto *or *Anopheles arabiensis*. Click here for file

Additional file 2 **Estimation of vectorial capacity**. For illustrative purposes, vectorial capacity was calculated for *A. gambiae *s.s. and *A. arabiensis *before and after scale-up of bed nets, using data from Asembo and Seme. Click here for file
