# Supplementary material for: Anopheles gambiae: historical population decline associated with regional distribution of insecticide-treated bed nets in western Nyanza Province, Kenya
Source: Malar J. 2010 Feb 26;9:62. doi: 10.1186/1475-2875-9-62 (PMC2838909; doi:10.1186/1475-2875-9-62)
Supplement: Additional file 1 — Historical records of Anopheles gambiae s.l. complex from indoor collections in western Kenya. List of references and summary of historical data used for composition of Figure 7, showing the year of sampling, village names or sample locations, number of houses sampled per location, and number of adult, female mosquitoes in the Anopheles gambiae sensu lato complex identified as either Anopheles gambiae sensu stricto or Anopheles arabiensis. [file 1475-2875-9-62-S1.doc]

Additional file 1

File format: DOC

Title: Historical records of *Anopheles gambiae* s.l. complex from indoor collections in western Kenya. Description: List of references and summary of historical and contemporary data used for composition of Figure 7, showing the year of sampling, village names or sample locations, number of houses sampled per location, and number of adult, female mosquitoes in the *Anopheles gambiae* sensu lato complex identified as either *Anopheles gambiae* *sensu stricto* or *Anopheles arabiensis*.

No. of female mosquitoes identified as:

Reference Year(s) of study No. villages or sites (no. house-collections) *A. gambiae* s.s. *A. arabiensis*

White [1]^1^ 1970 4 (28) 29 8

Service [2]^2^ 1971 12 (80) 191 48

Joshi *et al* [3]^3^ 1972 13 (4,460) 1,374 451

Highton *et al* (4)^4^ 1974-5 1 (not indicated) 52 1

Petrarca *et al* [5]^5^ 1986-7 2 (not indicated) 1,280 245

Taylor *et al* (6)^6^ 1988-9 4 (1,200) 977 115

Gimnig *et al* (7)^7^ 1996-7 1 (17,625) 7,928 503

Gimnig *et al* [8]^8^ 1997-98 15 (12,161) 261 16

Lindblade *et al* [9]^9^ 1999-2002 25 (2,120) 946 374

^1^Village names: Kanyamedha, Obambo, Ongalo, Tiengre. Method of collection: hand aspiration of females resting inside huts. Method of identification of sibling species: examination of ovarian polytene chromosome banding patterns.

^2^Village names: Barkorwa, Rata, Kajoake, N. Kaloha, Orando, Manuanda, N. Manuanda, Ngere, Kambuga, Koker, N. Ngere, S. Manuanda. Method of collection: hand aspiration of females resting inside huts. Method of identification of sibling species: examination of ovarian polytene chromosome banding patterns.

^3^Village names: Saradidi, Ramba, Kanyamedha, Tiengre, Kirmikaye, Oyenjre, Orando, Diemo, Kidianchei, Ngutu, Kadela, Runda, Nitienge. Method of collection: hand aspiration of females resting inside huts. Method of identification of sibling species: examination of ovarian polytene chromosome banding patterns.

^4^Village name: Chulaimbo. Method of collection: hand aspiration of females resting inside huts. Method of identification of sibling species: examination of ovarian polytene chromosome banding patterns.

^5^Village names: Kisian, Saradidi. Method of collection: hand aspiration of females resting inside huts (n = 1,475) and indoor pyrethrum spray catches (n = 50). Method of identification of sibling species: examination of ovarian polytene chromosome banding patterns.

^6^Village names: “4 contiguous villages near Asembo, on the shore of Lake Victoria, 50 km west of Kisumu at an elevation of 1,140 m.” Method of collection: hand aspiration of females resting inside huts. Method of identification of sibling species: species-specific ribosomal DNA probes hybridized to mosquito DNA in Southern blots.

^7^Village names: Raliew, Ongi'elo, Nguka, Aduoyo, Miyare, Wera, Ochuoga/Kowi, Abidha, Boi, Bwaja, Ndara, Katombo, Kaminogedo, Memba/Komenya, Mabinju, Ndwara, Nyangoma, Ujwanga/Nyachida, Mahaya. Method of collection: untreated bed net traps rolled up half way and hung over sleeping children. A total of 7,749 collections were made from 353 different houses in 1996, yielding 8,835 *A. gambiae* s.l.; while a total of 4,412 collections were made from 242 houses in 1997, yielding a total of 3,312 *A. gambiae* s.l. Method of identification of sibling species: polymerase chain reaction.

^8^Village names: Raliew, Ongi'elo, Nguka, Aduoyo, Miyare, Wera, Ochuoga/Kowi, Abidha, Boi, Bwaja, Ndara, Katombo, Kaminogedo, Memba/Komenya, Mabinju, Ndwara, Nyangoma, Ujwanga/Nyachida, Mahaya; and see Gimnig *et al* [8]. Method of collection: pyrethrum spray catches inside huts. Method of identification of sibling species: polymerase chain reaction.

^9^Village names: Three villages in Asembo and 22 villages in Seme are specified, but names were not given. Method of collection: pyrethrum spray catches inside huts. Method of identification of sibling species: polymerase chain reaction.

**References**

1. White GB: **The *Anopheles gambiae* complex and malaria transmission around Kisumu, Kenya.** *Trans R Soc Trop Med Hyg* 1972, **66**:572-581.

2. Service MW: *Identification of the Anopheles gambiae complex in the western Nyanza area, Kenya, 1971.* World Health Organization/Vector Biology and Control Unpublished Technical Report 1972, **72**.362.

3. Joshi GP, Service MW, Pradhan GD: **A survey of species A and B of the *Anopheles gambiae* Giles complex in the Kisumu area of Kenya prior to insecticidal spraying with OMS-43 (fenitrothion)**. *Ann Trop Med Parasitol* 1975, **69**:91-104.

4. Highton RB, Bryan JH, Boreham PFL, Chandler JA: **Studies on the sibling species *Anopheles* *gambiae* Giles and *Anopheles arabiensis* Patton (Diptera: Culicidae) in the Kisumu area, Kenya.** *Bull Ent Res* 1979, **69**:43-53.

5. Petrarca V, Beier JC, Onyango F, Koros J, Asiago C, Koech DK, Roberts CR: **Species composition of the *Anopheles gambiae* complex (diptera: Culicidae) at two sites in western Kenya**. *J Med Entomol* 1991, **28**:307-313.

6. Taylor KA, Koros JK, Nduati J, Copeland RS, Collins FH, Brandling-Bennett AD: ***Plasmodium* *falciparum* infection rates in *Anopheles gambiae*, *An. arabiensis*, and *An. funestus* in western Kenya**. *Am J Trop Med Hyg* 1990, **43**:124-129

7. Gimnig JE, Kolczak MS, Hightower AW, Vulule JM, Schoute E, Kamau L, Phillips-Howard PA: **Effect of permethrin-treated bed nets on the spatial distribution of malaria vectors in western Kenya**. *Am J Trop Med Hyg* 2003, **68**(suppl): 115-120.

8. Gimnig JE, Vulule JM, Lo TQ, Kamau L, Kolczak MS, Phillips-Howard PA, Mathenge EM, ter Kuile FO, Nahlen BL, Hightower AW: **Impact of permethrin-treated bednets on entomological indices in an area of intense year-round malaria transmission**. *Am J Trop Med Hyg* 2003, **68** (suppl):16-22.

9. Lindblade KA, Gimnig JE, Kamau L, Hawley WA, Odhiambo F, Olang G, ter Kuile Fo, Vulule JM, Slutsker L: **Impact of sustained use of insecticide-treated bednets on malaria vector species distribution and culicine mosquitoes**. *J Med Entomol* 2006, **43**:428-432.
