# Supplementary material for: Anopheles gambiae: historical population decline associated with regional distribution of insecticide-treated bed nets in western Nyanza Province, Kenya
Source: Malar J. 2010 Feb 26;9:62. doi: 10.1186/1475-2875-9-62 (PMC2838909; doi:10.1186/1475-2875-9-62)
Supplement: Additional file 2 — Estimation of vectorial capacity. For illustrative purposes, vectorial capacity was calculated for A. gambiae s.s. and A. arabiensis before and after scale-up of bed nets, using data from Asembo and Seme. [file 1475-2875-9-62-S2.doc]

Additional File 2

File format: DOC

Title: Estimation of vectorial capacity. Description: For illustrative purposes, vectorial capacity was calculated for both species before and after scale-up of bed nets, using data from Asembo and Seme. To do so, parity estimates are assumed to provide accurate estimates of the probability of daily survival, though this is strictly true only in the unlikely event of a stable age distribution. The interval for the gonotrophic cycle is estimated to be three days, and the Davidson formula is assumed to hold for the field conditions. The numbers of indoor resting mosquitoes was used as a proxy for man-biting rates, which were not measured directly here. The sporogonic cycle (EIP) was assumed to last 8 days. The human blood index (HBI) was the proportion of blood feeds on humans. With these limitations and assumptions, vectorial capacity for *A. gambiae s.s.* and *A. arabiensis* was estimated to decline by 99% one year after the 2006 roll out of bed nets. The estimates of vectorial capacity are useful for comparative purposes only and should not be interpreted in an absolute way. Here is the table of calculations:
